# Supplementary material for: The adeno-associated virus Rep proteins target PP4:SMEK1 by preventing substrate recruitment
Source: PLoS Pathog. 2026 Mar 10;22(3):e1014025. doi: 10.1371/journal.ppat.1014025 (PMC12994818; doi:10.1371/journal.ppat.1014025)
Supplement: S1 Methods — (DOCX) [file ppat.1014025.s001.docx]

**Supporting information – Methods**

**Generation of shRNA knockdown cell lines and treatment**

All primers and the shRNA oligos used in this section are detailed in the SI materials table. Oligos of the top and bottom strand of the shRNA targeting either the *PPP4C* 3’-UTR or the CDS of the firefly luciferase gene were ordered from Integrated DNA Technologies (IDT) and annealed to each other using the thermocycler (Biometra TOne) starting at 95 °C for 5 minutes, followed by a steady decrease in temperature during 45 minutes until 20 °C was reached. The annealed oligos were cloned in the AgeI and EcoRI linearized Tet-pLKO-puro backbone using NEBuilder HiFi DNA assembly by mixing equimolar ratios of both fragments. Correct ligation of the shRNA oligos in the Tet-On-pLKO backbone was verified through Sanger sequencing via LGC Genomics (Berlin, Germany). The Tet-On-pLKO-PPP4C-3’-UTR and Tet-On-pLKO-Luc-CDS plasmids were then used to produce lentiviral particles. 1*10^7^ HEK293T cells were seeded in a 10 cm dish followed by transient transfection with 4 μg pRSv REV, 2.5 μg pMD2 VsVg and 4 μg pMDLgag/pRRE #54 helper plasmids and 5 µg of the Tet-On-pLKO plasmid (detailed in SI materials table) using PEI Max. The cell culture medium was filtered three days post-transfection through a 0.22 μm filter and aliquots of the virus were stored at -80 °C until further use. Lentiviral titters were determined by ELISA. Transduction of HEK293T cells at a multiplicity of infection (MOI) of 25 was performed by seeding the cells at a confluency of 20 % in a 10 cm dish already containing the lentiviral particles pre-mixed with polybrene. Medium was changed 24 h post-transduction. Transduced cells were split and subjected to puromycin selection (1 μg/mL) three days post-transduction. The HEK293T shRNA PP4/Luc cell lines were stored in liquid nitrogen after four passages in the presence of puromycin. PP4 knockdown was induced by treating the cells with 1 μg/mL doxycycline for at least 72 h. Puromycin selection (1 μg/mL) was maintained during the experiments.

**Thermal shift assays**

Assays were performed to assess the stability of mutated LgBiT-SMEK1 in HEK293T cell lysate as described before. Briefly, 50 ﻿μL aliquots of cell lysates of HEK293T cells expressing LgBiT-SMEK1 WT or R557→A/E incubated for 5 minutes in a thermocycler (Biometra TOne) with a temperature gradient ranging from 45 to 69 °C. Next, 15 ﻿μL of each incubated aliquot was transferred to a white, non-binding 384-well plate (Greiner) already containing 5 ﻿μL assay buffer with ﻿100 μM furimazine. Thermal stability of the LgBiT-tagged proteins was determined by reading out the residual LgBiT luciferase activity using the Spark^®^ multimode microplate reader (Tecan Life Sciences).
