# Supplementary figures and images for: The adeno-associated virus Rep proteins target PP4:SMEK1 by preventing substrate recruitment
Source: PLoS Pathog. 2026 Mar 10;22(3):e1014025. doi: 10.1371/journal.ppat.1014025 (PMC12994818; doi:10.1371/journal.ppat.1014025)

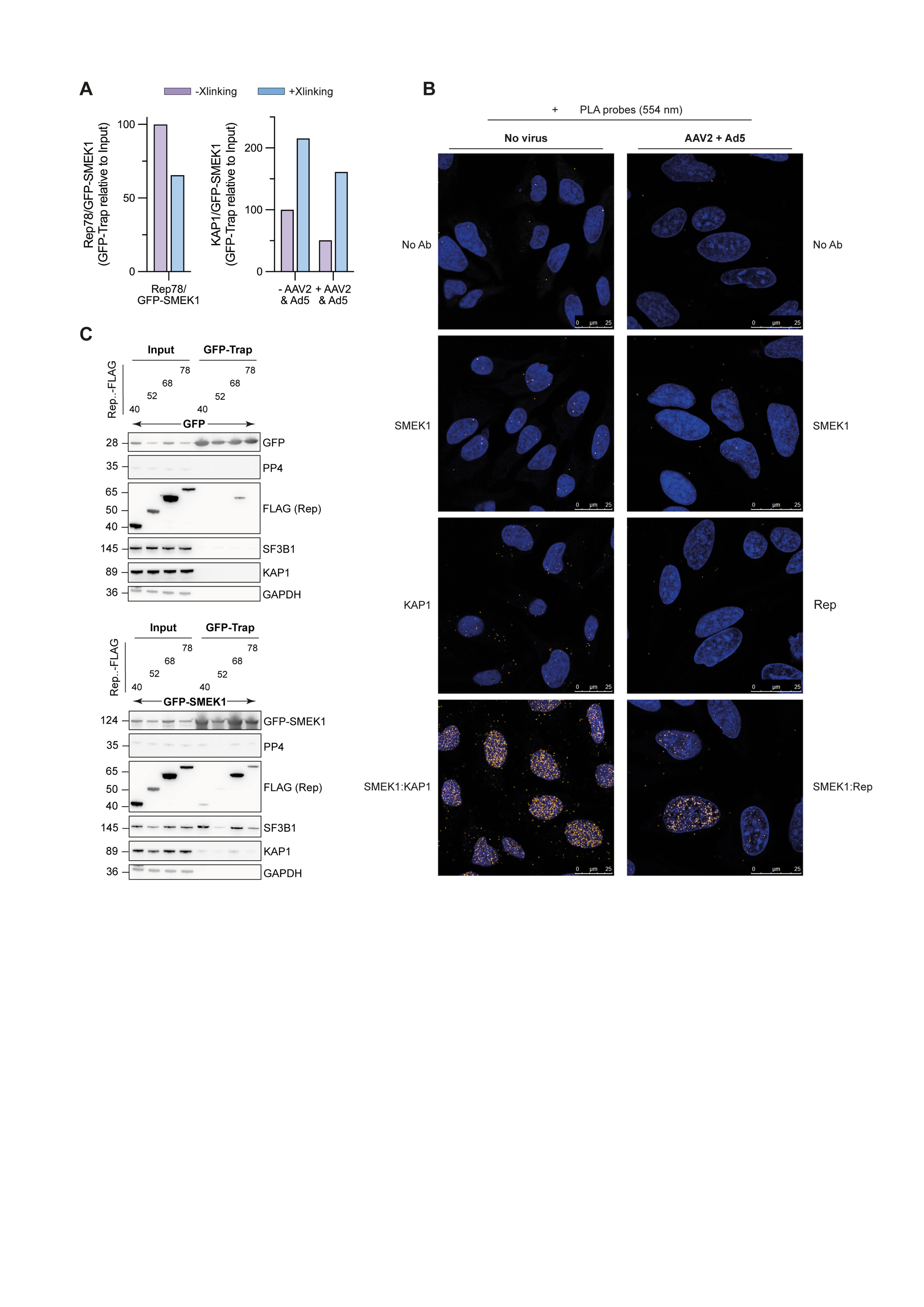

Supplement: S1 Fig — (A) Quantification of the immunoblot GFP-SMEK1, Rep78 and KAP1 band intensities both for the Input and GFP-trap conditions. Rep78/GFP-SMEK1 or KAP1/GFP-SMEK1 ratios of the GFP-trap conditions were divided by the same ratio of the corresponding input condition. Quantification was done for two independent experiments. (B) Proximity ligation assay (PLA) for the interaction between SMEK1:KAP1 in non-infected HeLa cells and for the SMEK1:Rep interaction in AAV2 (1000 IU) and Ad5 (MOI 5) co-infected HeLa cells. Cells incubated with either the single anti-SMEK1, -KAP1 or -Rep antibody, or no primary antibodies, served as a negative control. All conditions were incubated with the PLA probes. Orange dots indicate the association between SMEK1 and either KAP1 or Rep. Nuclei were counterstained with DAPI. Scale bar = 25 μm. (C) GFP-Trap experiment of GFP-tagged SMEK1 from cells ectopically expressing FLAG-tagged Rep (right panel). GFP expression alone served as a control (left panel). Input samples are shown on the left, while trap samples are shown on the right. (TIF) [file ppat.1014025.s003.tif]

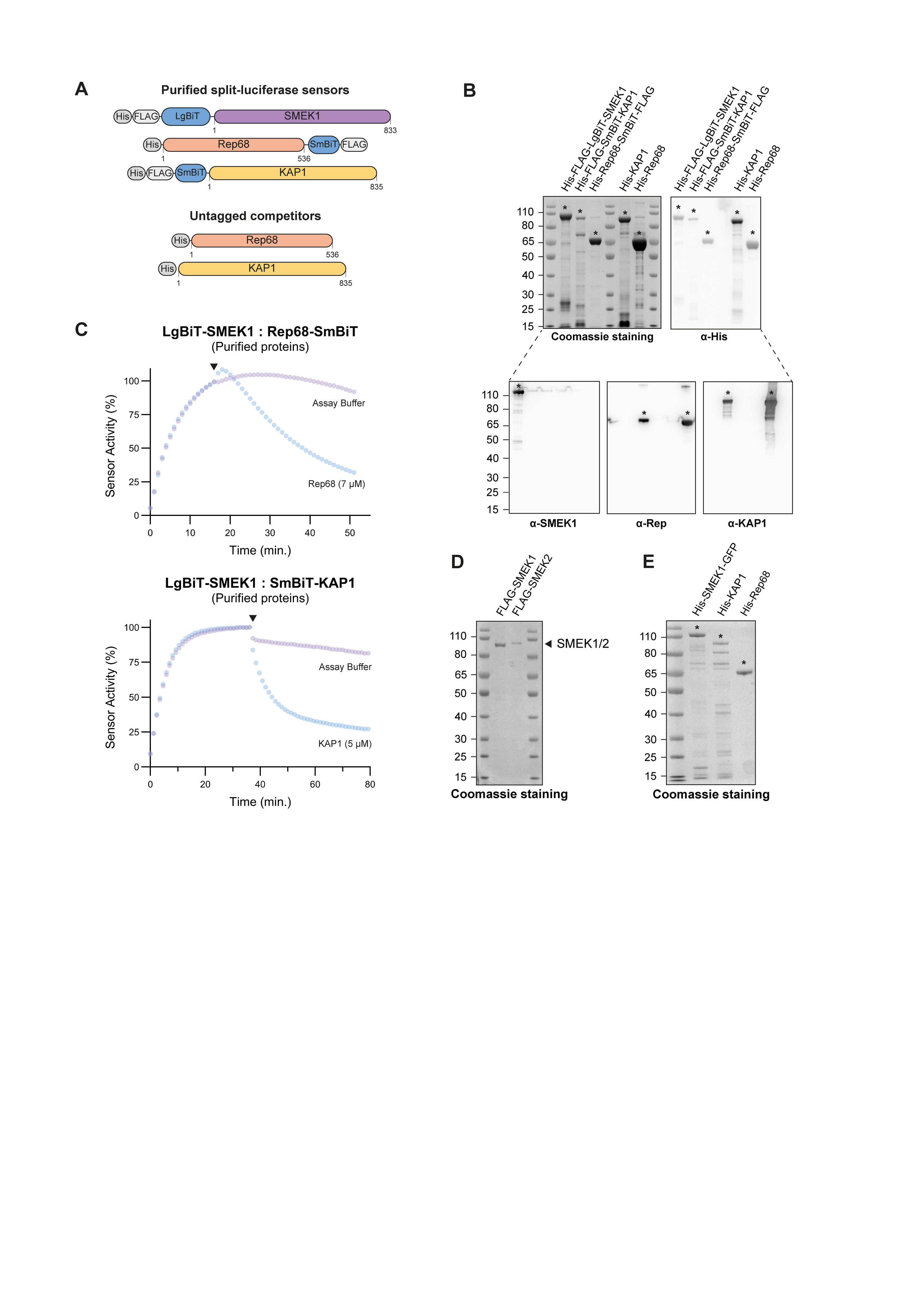

Supplement: S2 Fig — (A) Design of the split-luciferase sensors and untagged competitors used for the protein-protein interaction studies with purified proteins. Sensors and competitors were tagged N-terminally with a 10X His-tag for recombinant expression and purification from E. coli. (B) Coomassie staining and immunoblot visualization of the purified split-luciferase sensors and untagged competitors represented in S2A Fig and used in Figs 1E and S2C. The star indicates the respective species. (C) Kinetic-trace experiment with the LgBiT-SMEK1:Rep68-SmBiT and LgBiT-SMEK1:SmBiT-KAP1 purified interaction sensors. 0.5 nM LgBiT-SMEK1 was mixed with 10 nM of SmBiT-tagged protein. The black arrow indicates the addition of untagged competitor. Concentration of the competitors is indicated in the graph. The presented data is plotted as a percentage of the signal-to-background (S/B) ratio right before the addition of competitor. (D) Coomassie staining of FLAG-SMEK1 and FLAG-SMEK2 ectopically expressed in and purified from HEK293T cells. (E) Coomassie staining of the purified proteins used in the microscale thermophoresis (MST) assays shown in Fig 1G. (TIF) [file ppat.1014025.s004.tif]

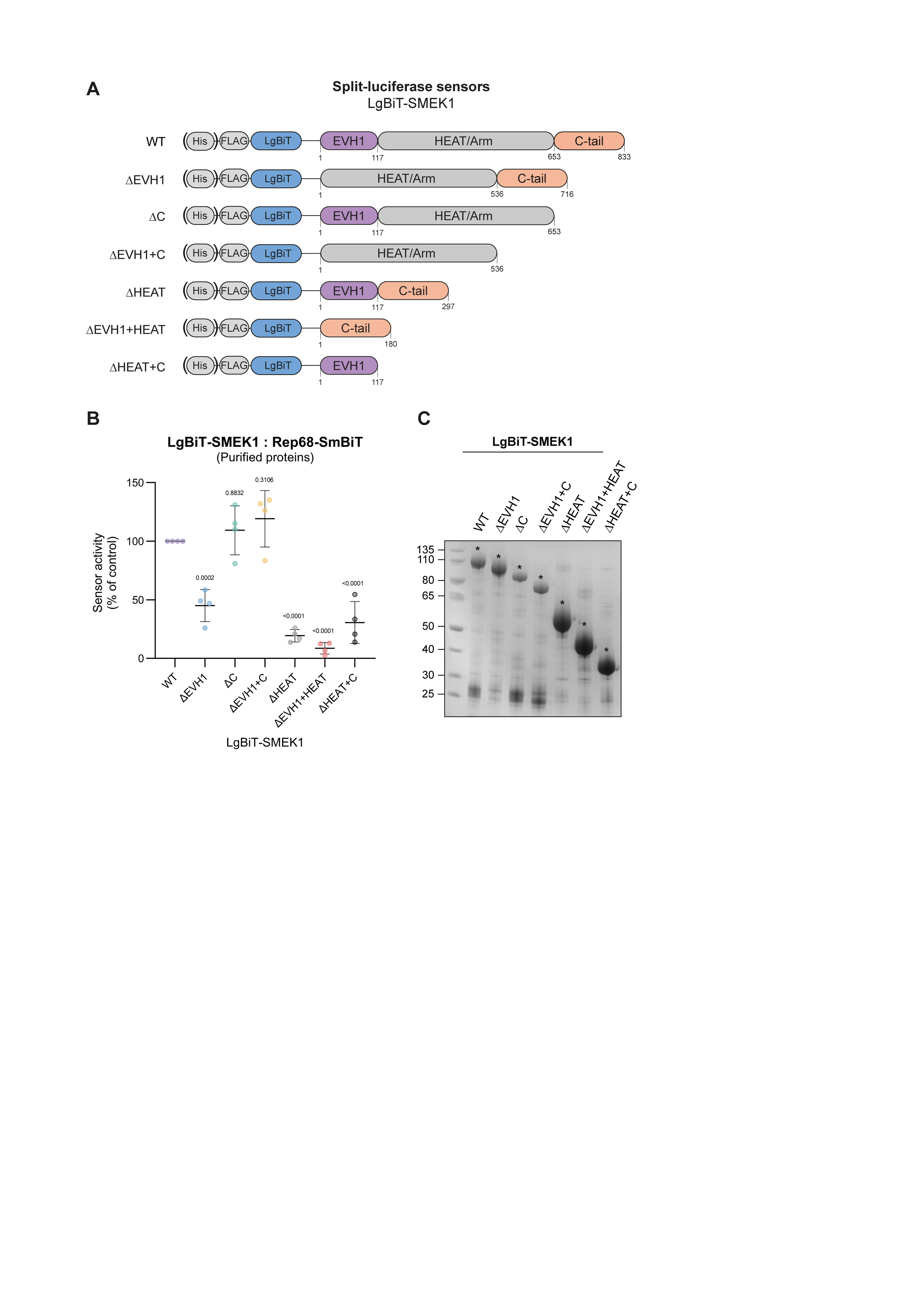

Supplement: S3 Fig — (A) Design of the truncated FLAG-LgBiT-SMEK1 split-luciferase sensors. Sensors were made for expression in HEK293T cells or E. coli (His-tagged). (B) Split-luciferase assays with the purified truncated LgBiT-SMEK1 (0.5 nM) and Rep68-SmBiT (10 nM) interaction sensors. End-point measurements were taken after mixing the sensor components and incubating them at room temperature for 20 minutes prior to read-out. Statistical significance was determined by an one-way ANOVA with Dunnett’s multiple comparison test. (C) Coomassie staining of the His-LgBiT-SMEK1 deletion mutants purified from E. coli. (TIF) [file ppat.1014025.s005.tif]

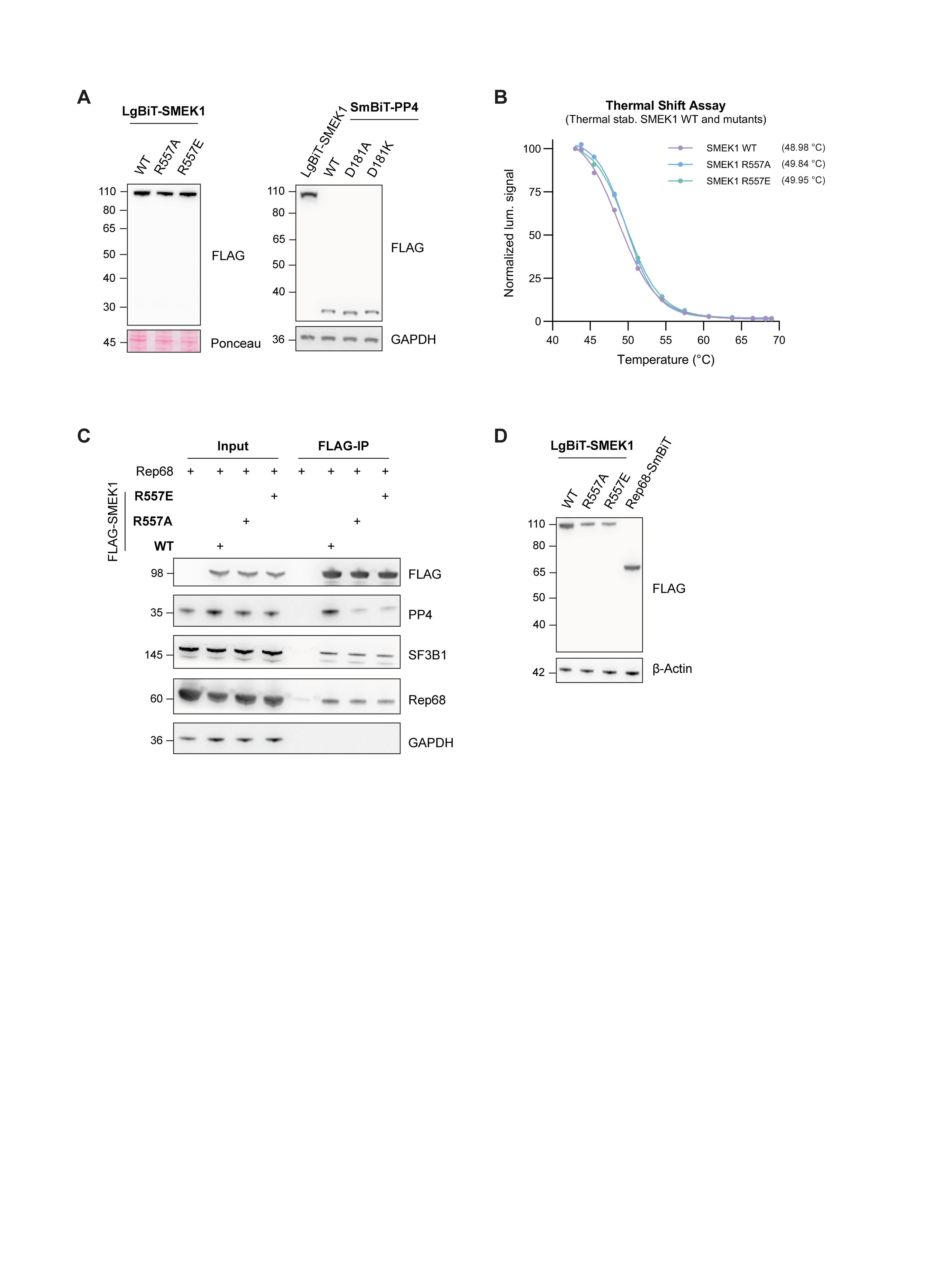

Supplement: S4 Fig — (A) Immunoblots of the split-luciferase lysates used in Fig 2F. (B) Thermal shift assay of WT and R557→A/E LgBiT-SMEK1 in lysates to assess the effect on the thermal stability of SMEK1 upon introduction of a point mutation in the HEAT/Arm domain. (C) FLAG-IP results of ectopically expressed FLAG-SMEK1 (WT and R557 mutants) showing loss of binding between endogenous PP4 and FLAG-SMEK1R557→A/E, while the interaction with ectopically expressed Rep68 and endogenously expressed SF3B1 is unaffected. (D) Immunoblot of the split-luciferase lysates used in Fig 2G. (TIF) [file ppat.1014025.s006.tif]

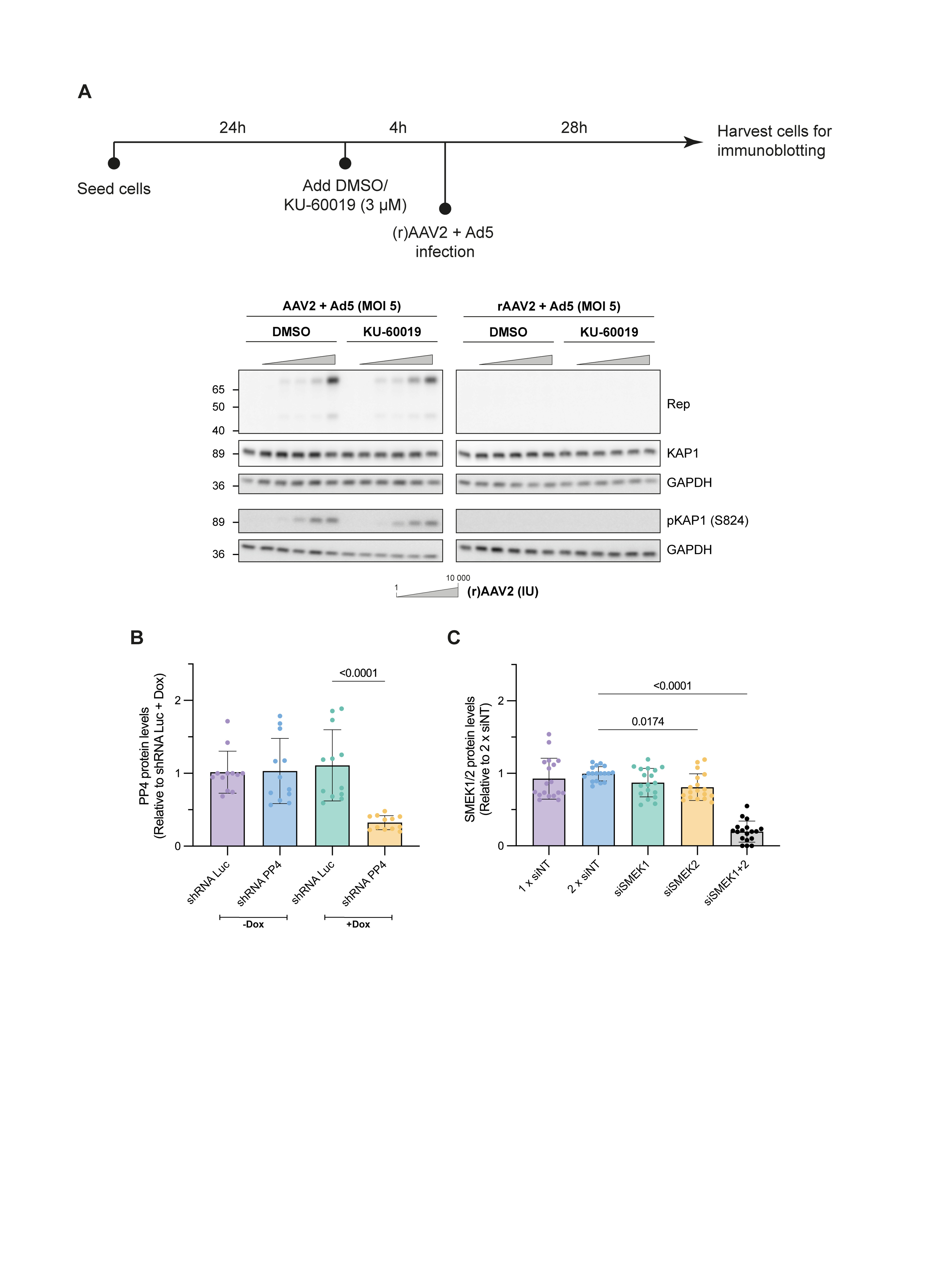

Supplement: S5 Fig — (A) HEK293T cells were pre-treated with a selective ATM inhibitor (KU-60019) (Cfin = 3 μM) prior to infection/transduction with increasing (r)AAV2 IUs and a fixed Ad5 MOI of 5. pKAP1S824 levels were assessed via immunoblotting 28h post-infection/transduction. (B) Quantification of the PP4 protein levels in the four main groups of the experiment shown in Fig 3B (mean ±SD; n = 12). Statistical significance was determined by an one-way ANOVA with Dunnett’s multiple comparison test. (C) Quantification of the SMEK1/2 protein levels in the five main groups of the experiment shown Fig 3D (mean ±SD; n = 18). Statistical significance was determined by an one-way ANOVA with Dunnett’s multiple comparison test. (TIF) [file ppat.1014025.s007.tif]

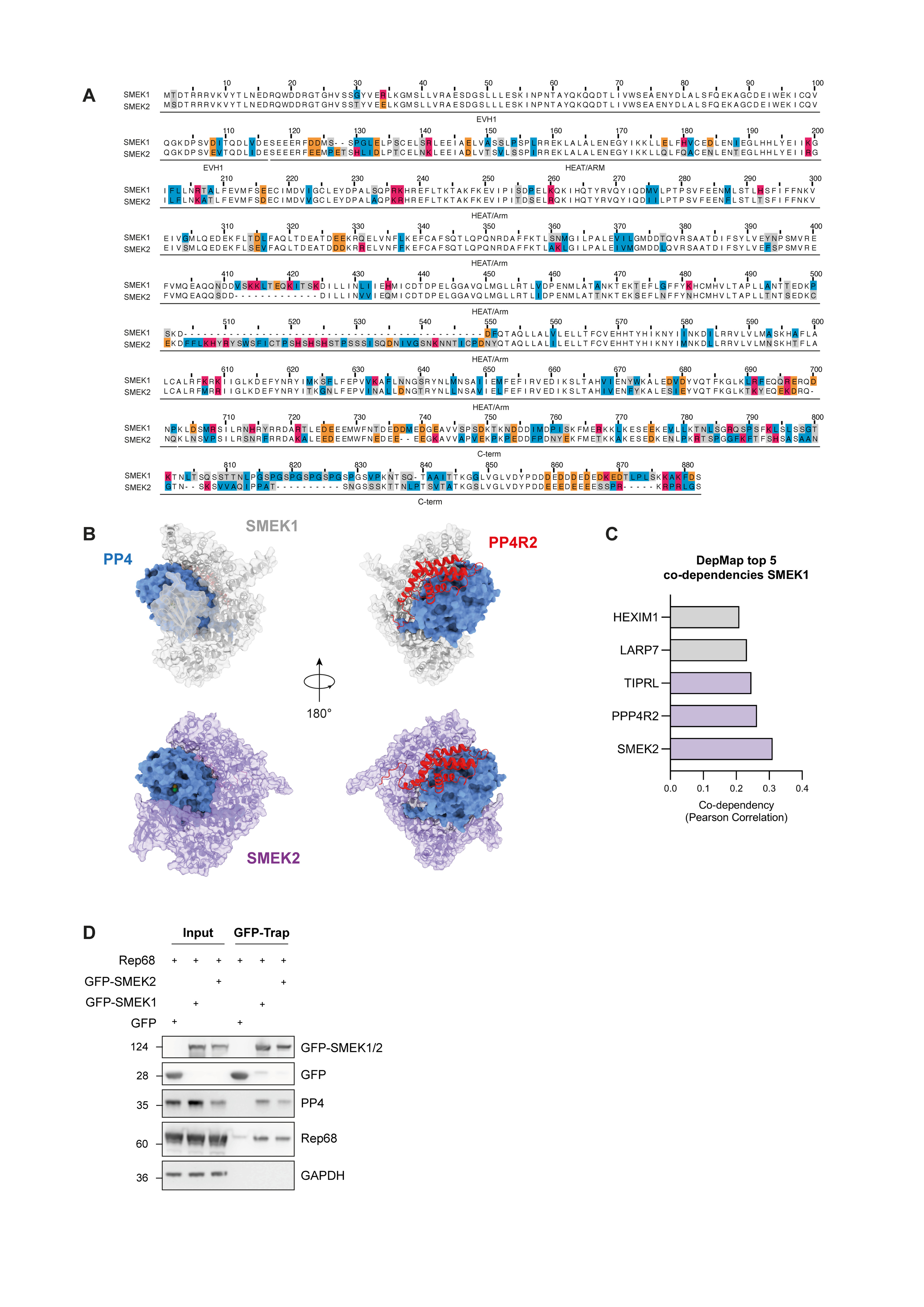

Supplement: S6 Fig — (A) Primary amino acid sequence alignment of SMEK1 and SMEK2, with only differing residues highlighted. Hydrophobic, negatively charged, positively charged, and other amino acids are color-coded in blue, orange, red, and grey, respectively. Sequence alignment was created with Jalview. (B) AlphaFold 3 multimer predictions of the PP4:PP4R2:SMEK1 and PP4:PP4R2:SMEK2 heterotrimeric complexes, showing the structural homology between the two complexes. (C) DepMap CRISPR knockout data showing a functional dependency between SMEK1 and SMEK2. (D) GFP-trap of GFP-tagged SMEK1 and SMEK2 from cells co-expressing Rep68. (TIF) [file ppat.1014025.s008.tif]

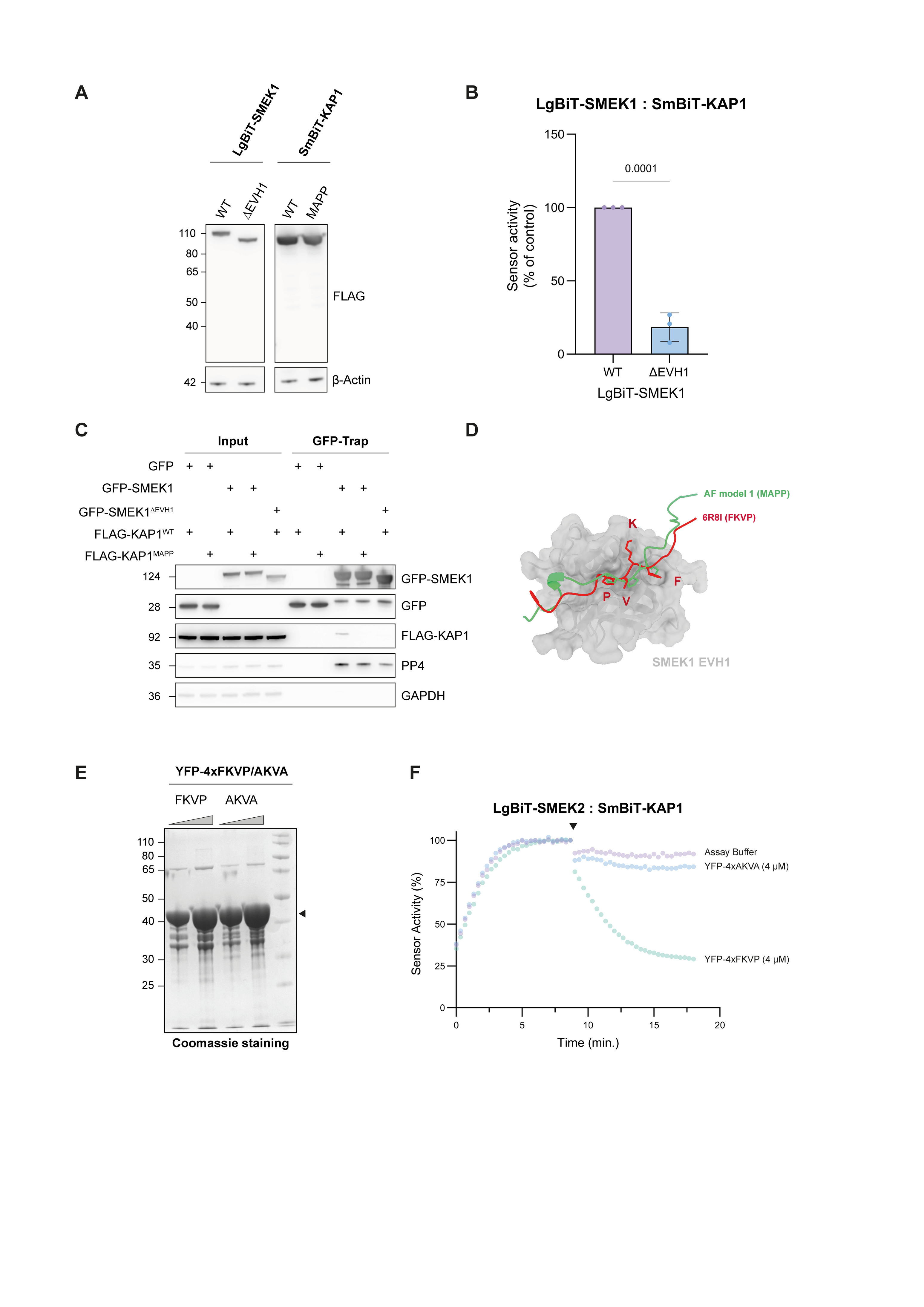

Supplement: S7 Fig — (A) Immunoblot of the LgBiT-SMEK1WT/ΔEVH1 and SmBiT-KAP1WT/MAPP split-luciferase lysates used in Figs 4B and S7B. (B) Lysate-based split-luciferase end-point measurement of the LgBiT-SMEK1WT:SmBiT-KAP1 and LgBiT-SMEK1ΔEVH1:SmBiT-KAP1 interaction sensors. Bioluminescence signal was read out after 25 minutes incubation at room temperature and plotted as a percentage of the LgBiT-SMEK1WT:SmBiT-KAP1 signal (mean ± SD; n = 3 independent repeats). Statistical significance was determined by a two-tailed unpaired t-test. (C) GFP-trap of ectopically expressed GFP-SMEK1 (WT and ΔEVH1) assessing the co-precipitation of ectopically expressed FLAG-KAP1 (WT and MAPP mutant). (D) Structural alignment of the EVH1:FKVP co-crystal structure (PDB 6R8I) with the AlphaFold 3 model 1 prediction shown in Fig 4C. (E) Coomassie staining of the purified YFP-4xFKVP and YFP-4xAKVA fusion proteins performed to assess the purity. (F) Kinetic-trace experiment of the LgBiT-SMEK2:SmBiT-KAP1 interaction sensor. Arrow indicates the addition of purified YFP-4xFKVP competitor or the AKVA control (concentrations indicated in the graph). The represented data is plotted as a percentage of the signal-to-background (S/B) ratio right before addition of the competitor. (TIF) [file ppat.1014025.s009.tif]

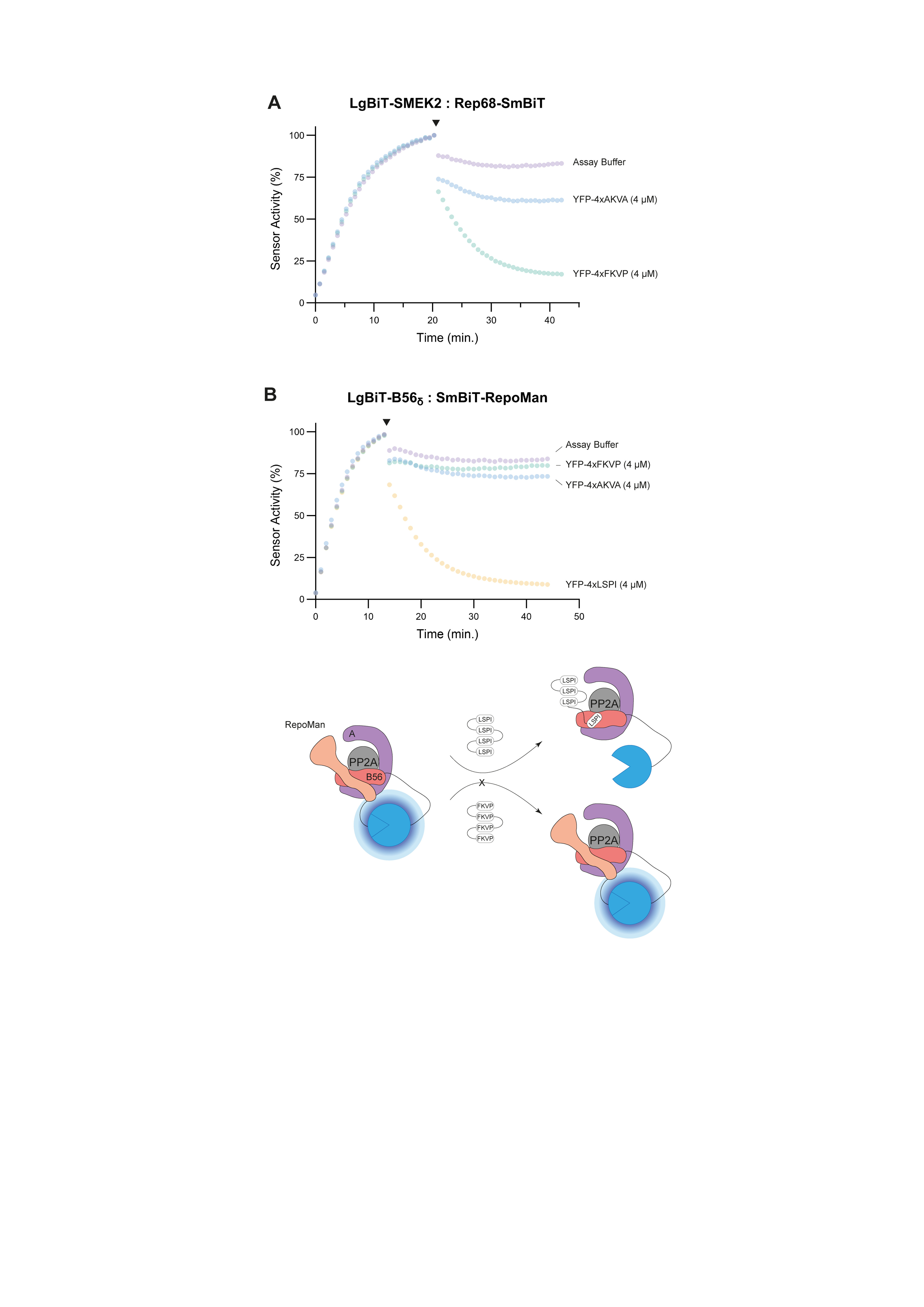

Supplement: S8 Fig — (A) Kinetic-trace experiment of the LgBiT-SMEK2:Rep68-SmBiT interaction sensor. Arrow indicates the addition of purified YFP-4xFKVP competitor or the AKVA control (concentrations indicated in the graph). The represented data is plotted as a percentage of the S/B ratio right before addition of the competitor. (B) Kinetic-trace experiment showing the time-dependent association of the LgBiT-B56δ:SmBiT-RepoMan split-luciferase sensor. The black arrow indicates the addition of the purified YFP-competitor peptides (concentration indicated in the graph). The represented data is plotted as a percentage of the S/B ratio right before the addition of the competitor. The data shown is a representative example of three independent repeats. (TIF) [file ppat.1014025.s010.tif]

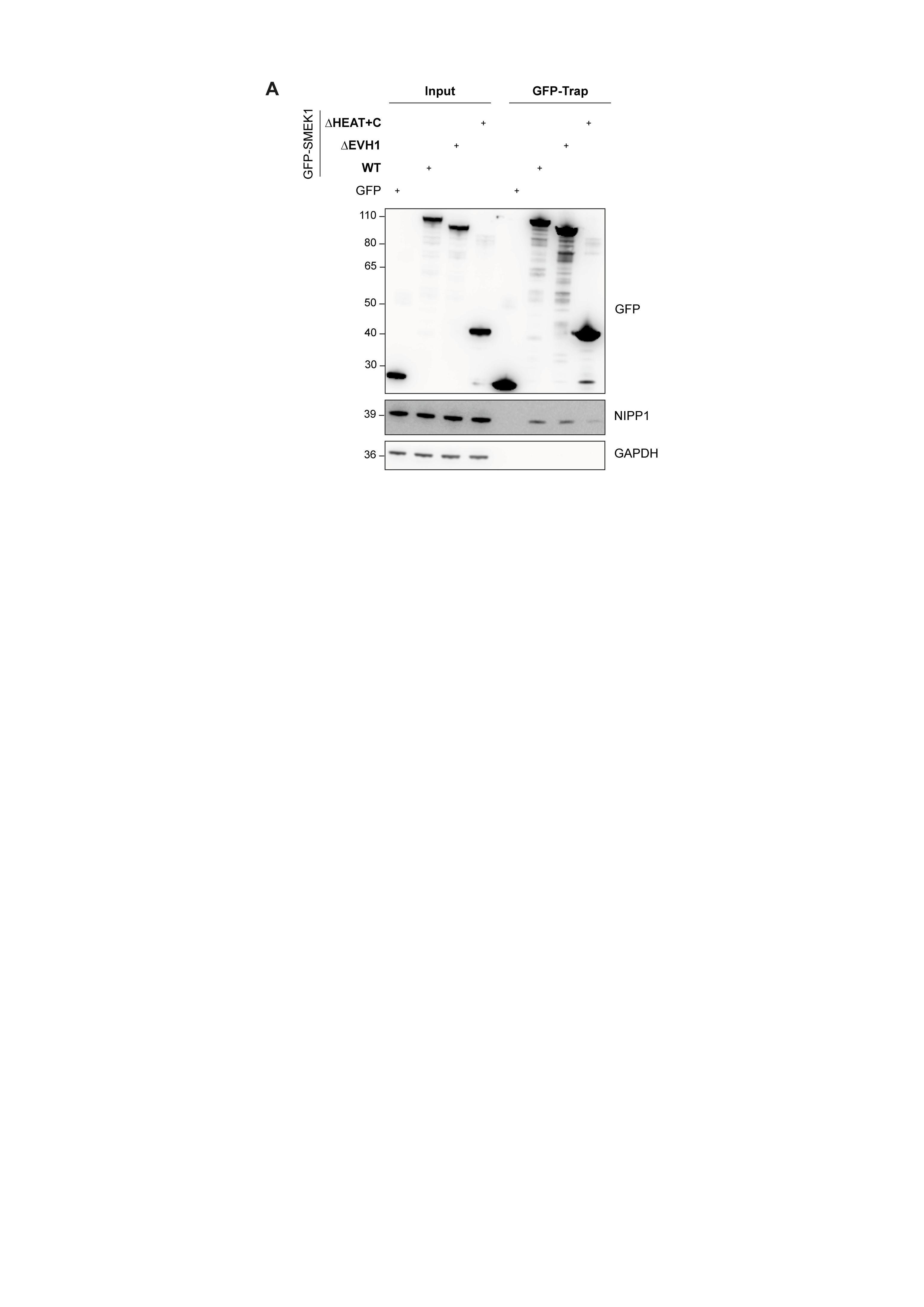

Supplement: S9 Fig — GFP-trap of GFP-tagged SMEK1 (WT, ΔEVH1, ΔHEAT+C-term) or GFP alone (control) to check for co-precipitation of endogenous NIPP1. (TIF) [file ppat.1014025.s011.tif]
