# Supplementary material for: The adeno-associated virus Rep proteins target PP4:SMEK1 by preventing substrate recruitment
Source: PLoS Pathog. 2026 Mar 10;22(3):e1014025. doi: 10.1371/journal.ppat.1014025 (PMC12994818; doi:10.1371/journal.ppat.1014025)
Supplement: Attachment — Submitted filename: Response to the reviewers.docx [file ppat.1014025.s013.docx]

**Response to the reviewers**

We sincerely thank all three reviewers for taking the time to carefully evaluate both our original and revised manuscript and for providing thoughtful and constructive feedback. We greatly appreciate their insightful comments, which have helped us improve the overall quality and clarity of our work. We believe that the additional experiments performed in response to their suggestions have further strengthened the support for our proposed hypothesis.

**Reviewer #1**

This study identifies the PP4:SMEK1/2 phosphatase complex as a key regulator of wild-type adeno-associated virus (AAV) replication. The authors show that AAV Rep68 binds SMEK1 to inhibit PP4 activity, leading to hyperphosphorylation of KAP1 and RPA2, which enhances viral gene expression and replication. They further describe how interactions between KAP1, SMEK1, and associated phosphatase complexes control this process, revealing a novel mechanism by which viruses modulate host phosphatases to promote viral replication.

The work is interesting and reveals a new mechanism in AAV-helpervirus-host cell interaction, but the study could be strengthened by adding important experimental controls.

Major Issues:

1. The authors use AAV2 Rep and Cap protein accumulation as a surrogate for viral replication. This should be validated at the level of viral DNA synthesis (e.g., Southern blot or qPCR of AAV genomes) and at the level of particle production (titration). Please provide these measurements (with replicates) or justify why the current readout is sufficient.

We thank the reviewer for this comment. Generally, AAV2 replication assays performed in our lab involve harvesting cells 28 hours post-infection and dividing them into two fractions. One fraction is utilized for protein extraction to quantify Rep protein expression (Fig. 3C and 3E). The second fraction is processed for gDNA extraction to quantify AAV2 viral genomes (vg) per cell via qPCR, utilizing a specific primer pair annealing to the *cap* ORF of the wild-type AAV2 genome (Fig. 3C and 3E). We know from experience that this assay provides a more accurate account of genome replication compared to the more qualitative Southern blot analysis.

As requested, we have strengthened the data set by including viral particle quantification by ELISA in the redesigned PP4 depletion experiments (see below). These ELISA results (n = 4) corroborate our viral DNA and protein expression quantifications (Fig. 3C).

Given the functional interdependence of PP4 and SMEK1/2, together with the consistent phenotypes observed throughout our study, we consider the VP quantification obtained in the PP4 knockdown experiments to be a reliable proxy for the SMEK1/2 knockdown condition. We therefore did not repeat the SMEK1/2 knockdown experiments for VP quantification, as we do not expect this to provide additional insight beyond what is already demonstrated.

2. Line 154–155: state explicitly how the apparent reduction of the SMEK1–Rep interaction after crosslinking was quantified (e.g., densitometry of specific bands normalized to input, number of biological replicates, statistical test). If quantification was performed, include the raw data and statistical summary.

We agree with the reviewer that the statement in lines 154-155 requires quantitative support to be fully interpretable. While our initial statement relied on qualitative visual assessment, we have now performed densitometric quantification of the specific bands (GFP-SMEK1, KAP1, and Rep78) from two independent biological replicates. In the revised analysis, Rep78/GFP-SMEK1 or KAP1/GFP-SMEK1 ratios of the pull-down samples were normalized to the same ratios of their respective input samples (Fig. S1A).

Although statistical power cannot be calculated with two replicates, the trends are consistent and distinct. We observed that formaldehyde crosslinking reduces the interaction of Rep78 with GFP-SMEK1, whereas the KAP1:GFP-SMEK1 interaction is stabilized/enhanced upon crosslinking. Importantly, we observed a reduction in overall KAP1:GFP-SMEK1 binding in the presence of Rep proteins, regardless of crosslinking. This finding further substantiates our central hypothesis that Rep proteins compete with KAP1 for SMEK1 binding. These quantifications have been added to Fig. S1A.

In addition to the pull-down experiments performed in the context of AAV2 replication, we included proximity ligation assays (PLA) in the supplementary figures (Fig. S1B). PLA assays were conducted on non-infected HeLa cells for the SMEK1:KAP1 interaction as a control, and on AAV2+Ad5 co-infected cells to visualize the SMEK1:Rep interaction. Appropriate negative controls were included, namely conditions without primary antibodies or with only a single primary antibody prior to incubation with the PLA probes, to assess potential false-positive PLA signals. As shown in Fig. S1B, all cells contained a robust PLA signal which is indicative for an interaction between endogenous SMEK1 and KAP1. Remarkably, for the SMEK1:Rep interaction, a strong PLA signal was only observed in a subset of cells, whereas the majority of cells displayed a low PLA signal. This observation likely reflects the heterogeneity of the AAV2+Ad5 co-infections. Overall, these PLA assays independently confirm the interactions of SMEK1 with both KAP1 and the Rep proteins, corroborating the GFP-Trap results in Figure 1B.

3. For the PP4 knockdown experiments, a cell line with doxycycline-inducible expression of a scrambled shRNA is a more appropriate control than an empty vector. Also, the experiment should be performed both in the presence and absence of doxycycline and knockdown efficiency and reproducibility (biological replicates) reported.

As requested, we redesigned the AAV replication assays in the shRNA PP4 knockdown (KD) cell line (hereafter referred to as shRNA PP4) and included the appropriate control conditions. The control HEK293T cell line now stably expresses a scrambled shRNA targeting firefly luciferase (shRNA Luc), which is not endogenously expressed in HEK293T cells. In line with the reviewer’s suggestion, replication assays were performed in both the shRNA Luc and shRNA PP4 cell lines in the presence or absence of doxycycline ([Dox] = 1 µg/mL) to exclude any potential effects of Dox treatment on AAV replication or viral gene expression. The resulting data, together with quantification of viral particle production (VP/µg protein), are presented in Fig. 3B and 3C. As requested, we also quantified the PP4 and SMEK1/2 KD efficiencies in Fig. 3B and 3D. Quantifications can now be found in Fig. S5B,C.

Based on the results from four independent biological replicates (n = 4), we conclude that PP4 depletion enhances AAV replication, viral gene expression, and viral particle production. Importantly, treatment of the shRNA Luc control cell line with 1 µg/mL Dox did not significantly affect AAV replication or gene expression compared to the untreated shRNA Luc condition. We note that the variability between independent experiments is relatively high. Such variability is frequently observed in AAV replication assays (Smith-Moore et al., 2018 PNAS). This likely reflects the heterogeneity in infection (see above), dependence of wild-type AAV on helper virus co-infection for successful replication and gene expression, as well as heterogeneity in PP4 or SMEK1/2 KD efficiency across cells during the assays. These factors are inherently difficult to fully control. However, the provided data clearly demonstrate that PP4:SMEK1/2 holoenzymes negatively regulate AAV processes.

Minor Issues:

1. Indicate the position of the FLAG-tags of the SmBit- and LgBit- constructs in the schematic illustrations.

We now indicated the position of the FLAG-tag in the schematic illustrations. The revised schematic representations can be found in Fig. 1C, S2A and S3A.

2. Explain the rationale for placing SmBit/LgBit at the chosen N- or C-termini of the tested proteins. If orientation could affect folding or interaction, provide data showing that tag position does not alter protein expression, localization, or binding (or include this as a caveat).

We based the design of the split-luciferase sensors and the orientation of the LgBiT- and SmBiT-tags on AlphaFold models as well as on personal communication with an established expert in the structural characterization of AAV Rep proteins. In addition, all SmBiT-tagged proteins have a 10 AA flexible linker between the tag and the protein, while LgBiT-tagged SMEK1 contains a 15 AA flexible linker. This was included to reduce steric hindrance with interacting proteins or protein complexes and to allow proper folding of the respective fusion protein.

The design of the SmBiT-PP4:LgBiT-SMEK1 interaction sensor was based on the AlphaFold 3 model shown in Fig. 2E. As has been well established, the C-terminus of the PP4 catalytic subunit is subject to methylation, which modulates its interaction with a subset of PP4 regulatory subunits (Lyons et al., 2021). In addition, the AlphaFold model consistently positioned the PP4 C-terminus within a groove of SMEK1. We therefore reasoned that fusion of either a large or small tag to the PP4 C-terminus would likely interfere with the formation of the PP4:SMEK1 holoenzyme complex, necessitating N-terminal tagging of PP4. For SMEK1, the tag needed to be positioned in close spatial proximity to the PP4 tag to allow efficient luciferase complementation. AlphaFold predictions indicated that the N-terminal EVH1 domain of SMEK1 is consistently located near the PP4 catalytic subunit. In contrast, the C-terminus of SMEK1 is predicted to be unstructured and was therefore considered less suitable for fusion with either the LgBiT or SmBiT tag. Based on these considerations, we chose to tag both PP4 and SMEK1 at their N-termini with the SmBiT and LgBiT tags, respectively.

For KAP1, AlphaFold modelling indicated that both the N- and C-termini are unstructured (Fig. 4A). Empirically, fusion of SmBiT to the N-terminus of KAP1 produced a robust bioluminescence signal when combined with LgBiT-SMEK1 containing HEK293T lysates. Based on this observation, we proceeded with the SmBiT-KAP1 fusion construct for subsequent experiments.

Finally, the SmBiT tag was fused to the C-terminus of Rep68. This design choice was guided by personal communication with Prof. Carlos R. Escalante, who advised that Rep modifications should preferentially be introduced at the C-terminus, as N-terminal tagging may interfere with proper protein folding.

We do not currently have experimental evidence indicating whether tagging affects the subcellular localization of the proteins. However, as the split-luciferase assays were performed using cell lysates rather than live cells, potential alterations in localization are unlikely to affect the interpretation of our results.

3. The Coomassie stain shown in Fig. S1C should be corroborated by immunoblotting for the relevant species to confirm band identity and loading.

We performed immunoblotting on the purified proteins used in Fig. 1E and S2C. The immunoblots were incubated either with an anti-His probe to simultaneously detect all His-tagged proteins, or with antibodies specific for SMEK1, KAP1, or Rep to visualize the individual proteins. We have added the data as Figure S2B.

4. Line 230: for clarity please revise the sentence to read: “The PP4-binding mutants of SMEK1 still interacted …”.

We changed this sentence as requested (Line 238).

5. Line 232: there is an inconsistency between the main text (“GFP-SMEK1”) and Fig. S3C (“FLAG-SMEK1”).

The mistake has been corrected.

6. For pull-down experiments where GFP is used as a control for GFP-SMEK fusions (e.g., Fig. 1B, Fig. 6B/C), using GFP fused to an unrelated protein of similar size would be a more appropriate control than GFP alone.

We thank the reviewer for this insightful comment. In our laboratory, GFP is routinely used as a control in GFP pull-down assays (e.g. Xiao et al., 2024 Nature Communications). GFP-tagged β-galactosidase (GFP-βGAL), however, is less commonly employed as a control. Given that the manuscript contains multiple GFP pull-down experiments, repeating all assays using an alternative GFP control would have been highly time-consuming. We therefore opted to address this concern by performing a control experiment (see figure below).

In this experiment, GFP, GFP-βGAL, and GFP-SMEK1 were immunoprecipitated from cells co-expressing Rep68 (see figure below). Co-precipitation of Rep68 and KAP1 was observed with GFP-SMEK1, whereas no detectable binding of these proteins was observed with either of the control constructs.

We believe that these results demonstrate that the GFP pull-down assays presented in the manuscript faithfully reflect the specific interactions under investigation.

7. The results shown in Fig. 4B should be validated by an independent biochemical approach (reciprocal pull-down or co-immunoprecipitation), with quantification of the interaction across replicates and appropriate negative controls.

The lysate-based split-luciferase readouts for the SMEK1^WT^:KAP1^MAPP^ (Fig. 4B) and SMEK1^ΔEVH1^:KAP1^WT^ (Fig. S7B) interactions were validated by GFP pull-down assays performed on cells ectopically expressing GFP-SMEK1^WT^ or GFP-SMEK1^ΔEVH1^ together with expression of either FLAG-KAP1^WT^ or FLAG-KAP1^MAPP^.

In contrast to the split-luciferase assays, no residual interaction with GFP-SMEK1 was detected in the pull-down experiments when the MAPP motif in FLAG-KAP1 was mutated to AAAA. Similarly, deletion of the SMEK1 EVH1 domain completely abolished the interaction with FLAG-KAP1^WT^ in this assay. These results indicate that mutation of the KAP1 MAPP motif or deletion of the SMEK1 EVH1 domain substantially reduces the binding affinity between both two proteins. Because of the lack of residual interaction, no exact quantifications could be performed.

It is well established that pull-down and co-immunoprecipitation assays can lack the sensitivity required to detect low-affinity protein-protein interactions, whereas split-luciferase assays have been shown both in literature and in our own experience to be well suited for detecting low affinity interactions. This difference in assay sensitivity likely explains the discrepancies observed between the split-luciferase (Fig. 4B and S7B) and GFP pull-down results (Fig. S7C). One representative experiment out of three independent repeats is shown in Figure S7C.

**Reviewer #2**

The manuscript presents a compelling biochemical model in which AAV Rep proteins directly interact with the PP4:SMEK1/2 phosphatase holoenzyme to inhibit substrate recruitment, thereby sustaining hyperphosphorylation of KAP1 and RPA2 to promote AAV replication. However, the functional genetic data provided to support this model, specifically the PP4 and SMEK1/2 knockdown experiments in Figures 3 are not sufficiently robust to substantiate the claimed biological significance.

Major Issues:

1. In Fig. 1F, the authors show co-precipitation of purified Rep68 and KAP1 with purified FLAG-SMEK1 (and SMEK2) using bead-based pulldown assays. However, these experiments are strictly qualitative and do not allow conclusions regarding binding affinity, stoichiometry, or specificity beyond binary association. Given that this interaction is a central mechanistic claim that AAV Rep68 directly binds SMEK1/2 and competes with cellular substrates, the manuscript would be significantly strengthened by quantitative biophysical measurements, such as SPR.

To address this, we performed microscale thermophoresis (MST) assays using the NanoTemper Monolith platform. This approach was recommended by the head of the VIB-KU Leuven Biophysics Platform, as surface plasmon resonance (SPR) instrumentation was not available and we already possessed a bacterial expression construct for GFP-tagged SMEK1 (SMEK1-GFP). MST is an immobilization-free biophysical technique that monitors changes in the movement of a fluorescently labelled protein upon localized irradiation by an infrared laser. Although less frequently used than SPR, MST has been shown to be a robust, versatile, and quantitative method for affinity determination (Wienken et al., 2010, Nature Communications).

In our assays, SMEK1-GFP served as the fluorescently labelled target at a final concentration of 225 nM. Determination of the binding affinities between SMEK1 and Rep68 or KAP1 proved challenging due to aggregation of Rep68 and KAP1 at higher concentrations. Nevertheless, we were able to derive dissociation constants (K_D_) that provide an approximate estimation of the interaction affinities, with K_D_ values of 453 ± 73 nM for Rep68 and 776 ± 224 nM for KAP1.

These affinities are consistent with our proposed model in which Rep68 competes with PP4:SMEK1 substrates such as KAP1 for binding to SMEK1. The MST-derived K_D_ values have now been included in Figure 1G and S2E.

2. The knockdown efficiencies for PP4C, SMEK1, and SMEK2 are clearly incomplete. As a result, the observed changes in Rep expression and AAV genome replication are modest (~2‑fold or less). In particular, the only two-fold in virus genome replication does not consider biologically significant. Furthermore, several comparisons show poor statistical significance (high P values). Given that the central model proposes that PP4:SMEK1/2 strongly suppresses AAV replication and that Rep68 exerts a dominant inhibitory effect on PP4, the current functional data do not demonstrate a robust biological requirement for PP4 or SMEK1/2.

We agree with the reviewer that the knockdown (KD) efficiencies of the targeted genes in the performed AAV replication assays are incomplete, and that a stronger depletion could potentially result in a more pronounced effect on AAV2 replication. To address this point, we attempted to further increase the KD efficiencies of PP4 (shRNA-mediated KD) and SMEK1/2 (siRNA-mediated KD) by performing titration experiments.

For PP4, KD of *PPP4C* in the HEK293T shRNA PP4 cell line is induced by doxycycline (Dox) treatment. We treated both the shRNA PP4 and the shRNA Luc (control) cell lines with increasing concentrations of Dox (0-4 µg/mL). Cells were harvested 96 h post-induction, corresponding to the time point of AAV2 and Ad5 co-infection in the AAV replication assays. However, using the current KD system, higher Dox concentrations did not result in further depletion of PP4 (see immunoblot and quantification below), and thus we were unable to achieve increased KD efficiencies. Of note, Dox concentrations greater then 1 μg/mL seemed to slow down cell proliferation in both cell lines, as observed by brightfield microscopy imaging (see below).

Similarly, we titrated increasing concentrations of siRNAs targeting SMEK1 and SMEK2 in HEK293T cells. As observed for PP4, these efforts did not result in improved KD efficiencies for either protein (see immunoblot and quantifications below). Moreover, transfection with higher siRNA concentrations, both for the NT and SMEK1/2 siRNA, consistently reduced cell viability, as evidenced by brightfield microscopy (see below). This reduction in viability is an important consideration for AAV replication assays, which critically depend on healthy cells. Introducing cellular stress may substantially affect AAV replication and could confound the interpretation of results by introducing effects that are independent of the intended gene depletion.

Minor Issues:

1. Quantification of the Western Blots in Fig. 3F is needed. Figure 3F presents qualitative blots showing increased phosphorylation of RPA2 and KAP1 with increasing AAV input. These data are consistent with the proposed model, but no quantification is provided.

We now quantified the results of three independent repeats. The corresponding graph can be found in Fig. 3G.

**Reviewer #3**

The authors describe here the interactions of AAV Rep proteins with PP4:SMEK1/2 and show that binding of Rep to this phosphatase blocks interaction and dephosphorylation of a known substrate, KAP1 which results in activation of the lytic cycle. They use a combination of assays to study the interaction, together with AlphaFold3 model prediction to guide the design of their mutants. Overall, this is a very interesting paper that show how viral proteins modulate phosphatase activity. However, my major concern is the lack of controls in expression levels when performing the split-luciferase experiments using cellular extracts.

Major Issues:

1. In all lysate-based split-luciferase assays the assumption is that all mutants/deletion mutants are expressed to the same level. However, this is not tested (or not shown). E.g. in Figure 2B the observed increase in interaction between SMEK1/deltaC or deltaEVH1+C with PP4 could this because this deletion mutant is expressed at much higher levels and therefore it appears that there is an increased interaction? Likewise, how well are the other deletion mutants express where you see a complete loss of binding? Expression of the LgBiT and SmBiT fusion constructs in panels E and F should also be shown to ensure that any observed phenotype is indeed due to a change in interaction/affinity rather than a change in expression levels. And for the mutant expressed in panel 4B- expression of both WT and mutant should be verified.

We agree with the reviewer that the expression levels of the various mutants tested in the split-luciferase assays should be verified by immunoblotting, as differences in bioluminescence signals could indeed arise from altered expression levels of the WT versus mutant proteins.

In our experimental setup, HEK293T cells overexpressing one component of the interaction sensor are lysed in 10 pellet volumes of assay buffer. Expression of all LgBiT- and SmBiT-tagged fusion proteins is routinely verified by immunoblotting for the common FLAG-tag fused to both the SmBiT and LgBiT-tag (see Fig. 1C, S2A and S3A). This approach allows us to assess relative expression levels and, when necessary, to normalize them by diluting the lysates with untransfected HEK293T lysate to achieve comparable protein levels to performing the assay.

These immunoblots were originally included in the supplementary information (Fig. S2B, S3A, S3D, and S6A). To improve clarity for the reader, we have now moved the immunoblot corresponding to Fig. 2B into the main figure as Fig. 2C. The remaining immunoblots have been retained in the supplementary information and are now more clearly referenced in the text to facilitate their identification by the readers.

Minor Issues:

1. In Figure 1B: does the Rep antibody used detect all 4 Rep proteins? Please give additional information in the figure legend.

We now implemented a sentence in the figure legend of Fig. 1B to indicate that the used Rep antibody recognizes the four different isoforms of Rep. The antibody used to visualize untagged Rep in this manuscript is the anti-AAV2 Replicase mouse monoclonal antibody, clone 303.9 from Progen (Cat # 61069).

2. Lines 153-156: “The enzyme-substrate interaction between SMEK1 and KAP1 was further enhanced by prior crosslinking, whereas the SMEK1:Rep interaction diminished upon crosslinking, possibly caused by stabilization of more dynamic SMEK1 protein complexes (Fig. 1B).” There are other possibilities why less protein is recovered when using crosslinkers, e.g. the epitope might be partly masked due to the crosslinking. Please, include as an alternative.

Upon closer inspection of the Rep protein levels in the input (Fig. 1B), we do not observe any difference between the crosslinked and non-crosslinked conditions. We agree with the reviewer that crosslinking can potentially affect immunoblot recovery by masking antibody epitopes. However, given the comparable input levels, we are confident that epitope masking does not account for the reduced Rep protein levels observed in the crosslinked GFP-Trap condition in our experiments.

3. Figure 1F: How does “purified” Flag-SMEK1 or 2 look like without PD. Can you detect them in Coomassie stained gel – how “clean” are they? Please include lanes where only Flag-SMEK1/2 are loaded.

We included a coomassie stain of purified FLAG-SMEK1 and -SMEK2 from HEK293T cells in Fig. S2D.

4. Fig. 3F – in the text (line 279) it is mentioned that increasing AAV in the presence of a fixed MOI of Ad5 is used, but in the figure it looks like both AAV and Ad5 are used in increasing amounts – please correct.

We added the MOI of Ad5 in Fig. 3F, as well as in Fig. 3A and S5A. We hope it is more clear now.

5. Line 623, typo, AAV2 rather than AA2.

The typo was corrected.

6. Figure legend. Figure 1B – Rep can’t be endogenous, it is a viral protein and therefore by definition is exogenous – please correct.

We have removed the word “endogenous” from the figure legend.

7. Figure 3A – wild-type and recombinant AAV2 is used but nowhere in the text is explained what the difference is between the two. Please add this in the text.

We now added a brief explanation of the difference in the text (Line 250-252)
